# Predictors of serum cobalamin and its association with homocysteine in community-dwelling older adults

**DOI:** 10.1038/s41430-018-0367-8

**Published:** 2018-12-04

**Authors:** Alexandra Jungert, Monika Neuhäuser-Berthold

**Affiliations:** Institute of Nutritional Science, Justus Liebig University, Goethestrasse 55, D-35390 Giessen, Germany

**Keywords:** Ageing, Nutrition, Epidemiology

## Abstract

**Background/objectives:**

This study investigates the predictors of serum cobalamin concentrations in community-dwelling older adults and the relationship between serum cobalamin and plasma homocysteine.

**Subjects/methods:**

Serum cobalamin and plasma homocysteine were measured by SimulTRAC-SNB radio assay and HPLC, respectively. Linear multiple regression analyses were performed with cross-sectional data of 352 participants aged 60–90 years to examine (1) the predictors of serum cobalamin and (2) the association between cobalamin and homocysteine status. Age, sex, body composition, diet, supplement use, smoking, serum folate, serum pyridoxal 5´-phosphate, serum creatinine, and selected diseases were considered as potential predicting/confounding factors.

**Results:**

Median values of serum cobalamin, plasma homocysteine, and dietary cobalamin intake were 256 pmol/L, 9.7 µmol/L, and 5.7 µg/day, respectively. In multiple regression analysis, cobalamin intake, sex, body composition, serum creatinine and smoking did not predict serum cobalamin (all *P* > 0.05). In contrast, age (*β* = 0.111, *P* = 0.031), serum folate (*β* = 0.410, *P* < 0.001) and diagnosis of chronic inflammatory bowel disease (IBD) (*β* = 0.101, *P* = 0.037) were positively and cancer diagnosis (*β* = −0.142, *P* = 0.003) was negatively associated with serum cobalamin. The model explained 23% of the variability of serum cobalamin. After exclusion of subjects with IBD/cancer diagnosis and/or vitamin B/multi-vitamin supplementation, only serum folate remained as positive predictor of serum cobalamin (*β* = 0.407, *P* < 0.001). Serum cobalamin was positively associated with inverse-transformed plasma homocysteine before (*β* = 0.298, *P* < 0.001) and after (*β* = 0.199, *P* < 0.001) multiple adjustments.

**Conclusions:**

Serum folate but not cobalamin intake or age proves to be a main predictor of cobalamin status. Nevertheless, independent of serum folate and other potential confounders, serum cobalamin is inversely associated with plasma homocysteine.

## Introduction

There is an ongoing debate on whether cobalamin intake should be increased in advanced age compared to younger ages. In light of this, higher reference values for older adults are discussed, as well as the necessity of cobalamin supplements in advanced age. This is based on observations that older individuals more often have an inferior cobalamin status [1–3], which might contribute to an age-related decline in cognitive function and an increased risk of cardiovascular diseases [4, 5]. The involvement of cobalamin in homocysteine metabolism might be one underlying mechanism of these associations.

The clinical relevance of a lower cobalamin status found in older subjects is unclear. First, the observed lower levels of cobalamin status parameters in these studies were not accompanied by classical hematological manifestations [6]; second, boundary values used to classify cobalamin deficiency or insufficiency are still under debate [3]; third, results regarding potential associations of either low serum cobalamin or cobalamin intake with cognitive decline [7–9] and cardiovascular diseases [10–13] are inconsistent.

Although pernicious anemia and severe atrophic gastritis are more prevalent in older than in younger subjects and cause clinical cobalamin deficiency [14], this relationship cannot be used to infer a higher cobalamin requirement of the general older population. In view of the current dietary reference values, insufficient cobalamin intake is rare in European adults including older subjects [15]. However, little is known on other potential determining factors of cobalamin status in community-dwelling older adults.

Therefore, the present study investigates whether dietary cobalamin intake is associated with serum cobalamin in older subjects independently of age, sex, body composition, supplement use, smoking behavior, serum folate, serum creatinine, and selected diseases. Furthermore, the association of serum cobalamin with plasma homocysteine is investigated by considering folate and vitamin B_6_ status.

## Methods

### Study population and design

Subjects were participants in a prospective cohort study, in which the nutritional and health status of community-dwelling senior citizens in Giessen, Germany (GISELA study) were investigated between 1994 and 2014 on multiple follow-ups. The study protocol was approved by the Ethical Committee of the Faculty of Medicine, Justus Liebig University, Giessen, Germany. All participants provided written informed consent. Recruitment was accomplished through notices at senior citizens’ meetings and medical practices, advertisements in local newspapers and subjects already participating. For enrollment, subjects had to be at least 60 years of age, physically mobile and available around Giessen on a long-term basis. Measurements took place after an overnight fast in the Institute of Nutritional Science in Giessen between July and October. A total of 587 subjects were enrolled but not all of the subjects participated in every follow-up.

The present analysis is based on cross-sectional data from the follow-up year 2002, which provided the largest sample size. From the 403 subjects participating in that year, subjects were excluded because of missing data on serum cobalamin (*n* = 3), body composition (*n* = 2), dietary intake (*n* = 34), general use of supplements/medicine (*n* = 18) and smoking behavior (*n* = 15). Two subjects declared non-fasting conditions and thus were not included in the analysis. The final study cohort consisted of 246 women and 106 men.

### Anthropometric data and body composition

Weight and height were assessed in light clothing without shoes via a calibrated scale with an integrated stadiometer, and body mass index (BMI) was calculated. A single-frequency (50 kHz) bioelectrical impedance analyzer (Akern-RJL BIA 101/S^®^; Data Input, Frankfurt, Germany) and the formula of Roubenoff et al. [16] were used to determine fat-free mass (FFM) and fat mass (FM).

### Dietary assessment and parameters related to lifestyle and health

Dietary intakes were determined using a 3-day estimated dietary record consisting of 146 food items and beverages, which was developed and validated for the GISELA study [17]. The nutrient and energy content of the food items was calculated by means of the German Food Code and Nutrition Data Base version 3.02 [18]. Nutrient intake was computed as individual mean values of the 3 days recorded. The use of B-vitamins and multi-vitamin supplements was assessed by means of two self-administered questionnaires and data of both questionnaires were combined to form the variable “vitamin B/multi-vitamin supplementation (no vs. yes)”. One of these questionnaires scrutinized the use of vitamin supplements during 3 days before blood sampling by brand name, frequency and dosage [19]. The other questionnaire assessed the overall use of vitamin B/multi-vitamin supplements. Total cobalamin intake represents the sum of dietary and supplementary cobalamin intake assessed by the above mentioned 3-day records divided by three to calculate daily intake.

Data on smoking behavior and medication were obtained by self-administered questionnaires. Smoking behavior was classified as a dichotomous variable in never-smokers vs. current/ex-smokers. Participants reported their diseases diagnosed by their physician using a questionnaire including predefined disease categories.

### Measurements in blood

After an overnight fast, venous blood was drawn into serum tubes, EDTA-containing tubes, and heparin-containing tubes. Plasma and serum aliquots were stored at −70 °C until analyzed. Serum cobalamin and folate were measured by SimulTRAC-SNB Radioassay Kit (MP Biomedicals, Eschwege, Germany). Plasma homocysteine was assessed by high performance liquid chromatography (HPLC) with fluorimetric detection using a modified method of Ubbink et al. [20]. Serum pyridoxal 5´-phosphate (PLP) was measured as indicator of vitamin B_6_ status by HPLC with fluorimetric detection using a modified method of Zempleni et al. [21]. Serum creatinine was analyzed using the Jaffé reaction [22]. Between-days CVs were 13.4% for cobalamin, 5.4% for folate, 1.5% for PLP, 3.3% for homocysteine and 3.2% for creatinine.

### Statistical analyses

Because some variables deviated from normal distribution (tested by Kolmogorov-Smirnov test), data were expressed as median and interquartile range if not stated otherwise. As serum cobalamin, folate and PLP, as well as dietary intake of nutrients were highly skewed, data underwent lg_10_ transformation. Plasma homocysteine underwent a reciprocal (inverse) transformation to approximate normally distributed residuals.

Descriptive characteristics of the participants were compared between two groups by Mann-Whitney *U* test when analyzing continuous variables and by chi-square test when analyzing categorical variables. Spearman correlation was used to investigate unadjusted associations of serum cobalamin and cobalamin intake with relevant factors. With the exception of dietary cobalamin intake, only those factors showing a trend for an association with serum cobalamin (*P* < 0.100) were considered in the subsequent linear multiple regression analysis with enter procedure to examine the predictors of lg_10_ serum cobalamin.

Linear multiple regression analysis using enter procedure with inverse transformed homocysteine as dependent variable and serum cobalamin as independent variable was performed with adjustments for age, sex, %FM, smoking behavior, lg_10_ serum folate, lg_10_ serum PLP, vitamin B/multi-vitamin supplementation, lg_10_ dietary intakes of cobalamin, folate and vitamin B_6_, as well as serum creatinine, lifetime diagnosis of cancer and chronic inflammatory bowel diseases (IBD). Analyses were performed using the statistical software package (SPSS 24.0, IBM, New York, USA). Significance level was set at *P* < 0.05 (two-sided).

## Results

Characteristics of the subjects are presented in Table 1. Women showed higher serum cobalamin and lower plasma homocysteine despite lower dietary cobalamin intake than men.

**Table 1:** Descriptive characteristics of the study population in 2002

	Entire cohort (*n* = 352)			Women (*n* = 246)			Men (*n* = 106)			
	Median	Percentiles		Median	Percentiles		Median	Percentiles		
		25	75		25	75		25	75	*P* ^a^
Age (years)	72.00	68.00	75.00	72.00	67.00	76.00	72.00	68.00	75.00	0.908
BMI (kg/m²)	26.79	24.40	29.66	26.85	24.34	29.93	26.58	24.63	29.38	0.713
FM (%)	40.19	33.36	44.68	42.80	38.65	46.33	30.38	27.40	34.38	<0.001
FFM (kg)	41.65	38.32	51.69	39.43	37.42	42.02	54.67	51.90	58.13	<0.001
Serum cobalamin (pmol/L)	255.53	176.15	364.11	263.44	184.27	383.35	239.38	168.39	310.20	0.039
Dietary cobalamin intake (µg/day)	5.73	4.32	7.91	5.59	4.25	7.46	5.98	4.72	8.58	0.011
Total cobalamin intake (µg/day)^b^	6.06	4.66	8.31	5.99	4.49	7.88	6.72	4.87	9.80	0.025
Serum folate (nmol/L)	18.39	12.19	26.63	18.83	12.21	27.75	16.68	11.15	24.12	0.101
Dietary total folate intake (µg/day)	242.18	198.93	300.16	240.30	197.17	298.57	245.21	199.67	302.31	0.490
Serum PLP (nmol/L)	39.00	25.00	58.00	38.50	25.00	58.00	39.00	26.00	59.00	0.568
Dietary vitamin B_6_ intake (mg/day)	1.52	1.19	1.86	1.48	1.17	1.82	1.58	1.25	2.07	0.062
Plasma homocysteine (µmol/L)	9.69	8.16	11.86	9.36	8.04	11.61	10.05	8.74	12.22	0.022
Serum creatinine (mg/100 mL)^c^	0.96	0.89	1.06	0.93	0.85	1.01	1.05	0.96	1.16	<0.001

		*n*	%		*n*	%		*n*	%	
Current/past smokers		136	38.6		63	25.6		73	68.9	<0.001
Vitamin B/multi-vitamin supplement users		111	31.5		82	33.3		29	27.4	0.268

*Note*: Data are presented as median, 25th and 75th percentiles for continuous variables and absolute and relative frequencies for categorical variables

^a^Mann-Whitney *U* test and chi-square test for analyzing sex differences

^b^Twenty-two subjects had missing data on quantitative cobalamin intake by supplements within three days before blood sampling. Therefore, total cobalamin intake could not be calculated for these subjects

^c^Eleven subjects had missing creatinine measurements

**Fig. 1:**
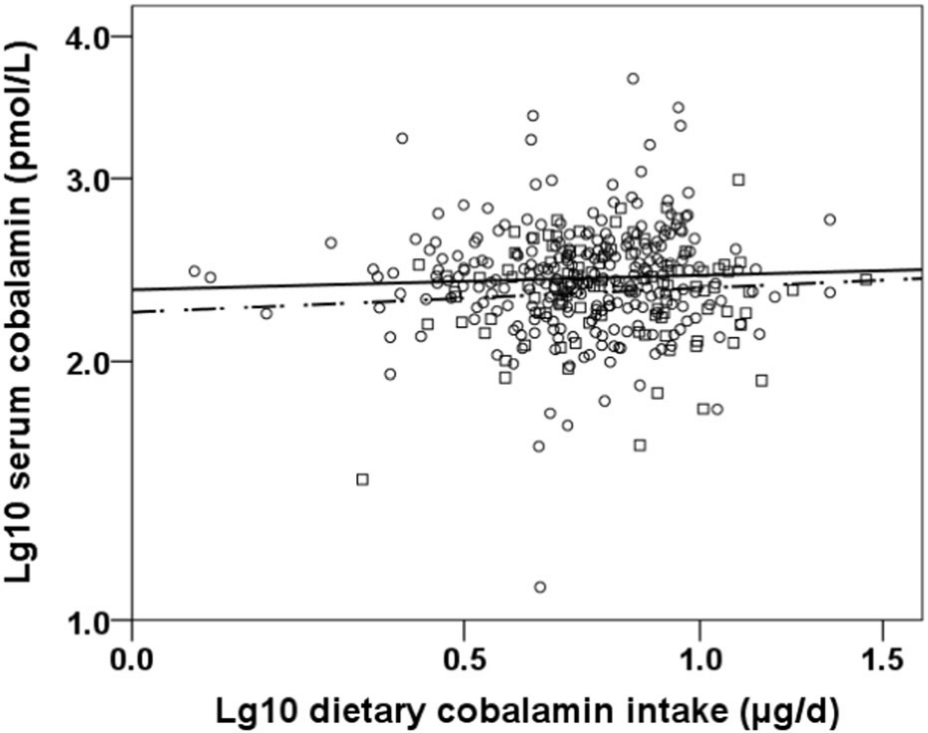
Association between lg_10_ dietary cobalamin intake and lg_10_ serum cobalamin stratified by sex (solid line and (○) open circle represent women; broken line and (□) open square represent men)

Serum cobalamin, plasma homocysteine and dietary cobalamin intake ranged between 12.8 and 4767 pmol/L, 3.8 and 30.9 µmol/L, and 1.2 and 28.2 µg/day, respectively. Serum cobalamin ≤ 148 pmol/L was present in 17% of the subjects, and 20% showed a dietary cobalamin intake < 4.0 µg/day, with no sex differences in this respect (*P* > 0.05). Subjects with serum cobalamin ≤ 148 pmol/L vs.> 148 pmol/L showed equal dietary cobalamin intakes [5.9 (4.4–7.9) vs. 5.7 (4.3–7.9) μg/day, *P* = 0.777]. Likewise, serum cobalamin did not differ between subjects with dietary intake levels < and ≥ 4.0 µg/day [264 (180–332) vs. 253 (174–370) pmol/L, *P* = 0.702]. Vitamin B/multi-vitamin supplement users had higher serum cobalamin than non-users [308 (219–433) vs. 237 (160–339) pmol/L, *P* < 0.001]. The unadjusted association between lg_10_ dietary cobalamin intake and lg_10_ serum cobalamin stratified by sex is illustrated in Fig. 1.

Subjects diagnosed with stomach disease (*n* = 67), liver/gall bladder disease (*n* = 64) or urinary tract/kidney disease (*n* = 15) until 2002 did not differ in serum cobalamin from subjects without such a disease (all *P* > 0.05). Subjects with cancer diagnosis (*n* = 44) had lower serum cobalamin than those not reporting cancer diagnosis [204 (128–298) vs. 259 (186–373) pmol/L, *P* = 0.003], but had equal dietary cobalamin intakes [6.1 (4.5–9.7) vs. 5.7 (4.3–7.7) µg/day, *P* = 0.119]. Subjects with IBD (*n* = 10) had higher serum cobalamin [320 (273–574) vs. 252 (174–363) pmol/L, *P* = 0.040] and higher dietary cobalamin intake [8.0 (5.9–11.4) vs. 5.7 (4.3–7.9) µg/day, *P* = 0.022] than subjects without IBD. Subjects who reported to use stomach medicine (*n* = 41) did not differ in serum cobalamin [251 (188–355) vs. 256 (174–369) pmol/L, *P* = 0.849] or dietary cobalamin intake [5.8 (4.3–7.1) vs. 5.7 (4.3–7.9) µg/day, *P* = 0.797] from subjects who reported not to use these medications.

The results of the Spearman correlation are given in Table 2. Serum cobalamin correlated with total cobalamin intake, sex, smoking, vitamin B/multi-vitamin supplementation, diagnosis of cancer and IBD, consumption of milk/milk products and serum folate. Dietary intake of cobalamin correlated with sex, age, FFM, diagnosis of IBD and consumption of milk/milk products, meat/meat products, eggs, fish and alcoholic beverages. Current/past smokers showed lower serum cobalamin compared with never smokers [239 (173–316) vs. 263 (184–397) pmol/L, *P* = 0.018], whereas cobalamin intakes were almost identical [5.7 (4.3–7.9) vs. 5.7 (4.3–7.9) µg/day, *P* = 0.617]. In sex-specific analysis, no differences in serum cobalamin according to smoking behavior were evident (*P* > 0.05).

**Table 2:** Spearman correlations to find variables associated with serum cobalamin and dietary cobalamin intake (*n* = 352)

	Serum cobalamin (pmol/L)		Dietary intake of cobalamin (µg/day)	
	*r*_*Rho*_	*P*	*r*_*Rho*_	*P*
Dietary cobalamin intake (µg/day)	0.019	0.728		
Total cobalamin intake (µg/day)^a^	0.112	0.042	0.896	<0.001
Sex (female vs. male)	−0.110	0.039	0.135	0.011
Age (years)	0.074	0.166	0.190	<0.001
FFM (kg)	−0.094	0.079	0.134	0.012
Current/past smoking (no vs. yes)	−0.126	0.018	0.027	0.618
Vitamin B/multi-vitamin supplements (no vs. yes)	0.231	<0.001	−0.081	0.130
Consumption of milk/milk products (g/day)	0.160	0.003	0.304	<0.001
Consumption of meat/meat products (g/day)	0.050	0.350	0.468	<0.001
Consumption of eggs (g/day)	−0.023	0.674	0.125	0.019
Consumption of fish (g/day)	0.054	0.309	0.541	<0.001
Consumption of alcohol free beverages (g/day)	0.067	0.211	0.056	0.298
Consumption of alcoholic beverages (g/day)	−0.025	0.639	0.108	0.042
Serum creatinine (mg/100 mL)^b^	−0.016	0.769	0.036	0.511
Serum folate (nmol/L)	0.470	<0.001	−0.071	0.182

^a^Twenty-two subjects had missing data on quantitative cobalamin intake by supplements within 3 days before blood sampling

^b^Eleven subjects had missing creatinine measurements

The results of the linear multiple regression analyses are presented in Table 3. Age, lg_10_ serum folate, as well as diagnosis of cancer and IBD were significant predictors of lg_10_ serum cobalamin. The regression model explained 23% of the variance in lg_10_ serum cobalamin. After nine subjects with extreme cobalamin concentrations (12.8 and ≥ 1000 pmol/L) were excluded to approximate normal distribution of residuals, similar results were found, except for the age effect, which became non-significant, and an increase in the explained variance to 28% (data not shown). The results remained unchanged when lg_10_ dietary cobalamin intake (µg/day) was replaced by consumption of milk/milk products (g/day) or by lg_10_ cobalamin intake via milk/milk products (µg/day) (data not shown). Furthermore, when lg_10_ dietary cobalamin intake was replaced by lg_10_ total cobalamin intake, the results were equal (Table 3). When subjects diagnosed with IBD or cancer and/or use of vitamin B/multi-vitamin supplements were excluded, only lg_10_ serum folate was significantly associated with lg_10_ serum cobalamin (Table 3).

**Table 3:** Multiple regression analyses to identify predictors of lg_10_ serum cobalamin

	Entire cohort (*n* = 352)^a^			Subjects with detailed information on cobalamin intake by supplements (*n* = 330)^b^			Subjects without vitamin B/multi-vitamin supplements, cancer or IBD diagnosis (*n* = 205)^c^		
	Lg_10_ serum cobalamin (pmol/L)			Lg_10_ serum cobalamin (pmol/L)			Lg_10_ serum cobalamin (pmol/L)		
	*B*	*β*	*P*	*B*	*β*	*P*	*B*	*β*	*P*
Intercept	1.297		< 0.001	1.246		< 0.001	1.519		< 0.001
Age (years)	0.005	0.111	0.031	0.006	0.119	0.023	0.003	0.065	0.352
Sex (female vs. male)	−0.083	−0.133	0.160	−0.082	−0.133	0.180	−0.058	−0.100	0.447
FFM (kg)	0.003	0.099	0.302	0.004	0.106	0.287	0.002	0.051	0.700
Lg_10_ serum folate (nmol/L)	0.439	0.410	< 0.001	0.442	0.410	< 0.001	0.457	0.407	< 0.001
Current/past smoking (no vs. yes)	−0.032	−0.055	0.292	−0.034	−0.058	0.287	−0.018	−0.033	0.646
Lg_10_ dietary cobalamin (µg/day)	0.077	0.053	0.278				0.058	0.042	0.534
Lg_10_ total cobalamin intake (µg/day)				0.081	0.065	0.199			
Use of vitamin B/multi-vitamin supplements (no vs. yes)	0.031	0.050	0.336	0.017	0.027	0.624			
Lifetime diagnosis of cancer (no vs. yes)	−0.123	−0.142	0.003	−0.136	−0.154	0.002			
Lifetime diagnosis of IBD (no vs. yes)	0.173	0.101	0.037	0.177	0.105	0.034			
corr. R^2^		0.231			0.232			0.154	

^a^Linear multiple regression analysis with lg_10_ serum cobalamin concentrations as dependent variable and sex (female vs. male), age (years), FFM (kg), lg_10_ dietary intake of cobalamin (µg/day), use of vitamin B/multi-vitamin supplements (no vs. yes), smoking behavior (never vs. current/past smoking), cancer diagnosis (no vs. yes), IBD diagnosis (no vs. yes) and lg_10_ serum folate (nmol/L) as independent variables. Data are shown as non-standardized coefficient beta (*B*), standardized coefficient beta (*β*) and adjusted coefficient of determination (corr. R²)

^b^Linear multiple regression analysis with lg_10_ serum cobalamin concentrations as dependent variable and sex (female vs. male), age (years), FFM (kg), lg_10_ total cobalamin intake (µg/day), use of vitamin B/multi-vitamin supplements (no vs. yes), smoking behavior (never vs. current/past smoking), cancer diagnosis (no vs. yes), IBD diagnosis (no vs. yes) and lg_10_ serum folate (nmol/L) as independent variables. Because 22 participants had missing data on quantitative cobalamin intake by supplements, the sample size was reduced to 330 subjects in this analysis

^c^Linear multiple regression analysis with lg_10_ serum cobalamin concentrations as dependent variable and sex (female vs. male), age (years), lg_10_ dietary intake of cobalamin (µg/day), FFM (kg), smoking behavior (never vs. current/past smoking) and lg_10_ serum folate (nmol/L) as independent variables

In linear regression analyses, lg_10_ serum cobalamin was positively associated with inverse-transformed plasma homocysteine before (*B* < 0.001, *β* = 0.298, *P* < 0.001) and after (*B* < 0.001, *β* = 0.199, *P* < 0.001) adjustments for age, sex, %FM, smoking behavior, lg_10_ serum folate, lg_10_ serum PLP and use of vitamin B/multi-vitamin supplements. The results remained unchanged when lg_10_ intakes of cobalamin, folate and vitamin B_6_, as well as serum creatinine and diagnosis of cancer and IBD were considered as additional covariables (*B* = 0.024, *β* = 0.233, *P* < 0.001). In this analysis, the sample size was reduced to 341 subjects because of missing values for serum creatinine. After excluding subjects with serum cobalamin ≥ 1000 pmol/L (*n* = 8), lg_10_ serum cobalamin was still associated with inverse-transformed plasma homocysteine before (*B* = 0.031, *β* = 0.269, *P* < 0.001) and after (*B* = 0.014, *β* = 0.122, *P* = 0.026) adjustments for age, sex, %FM, smoking behavior, lg_10_ serum folate, lg_10_ serum PLP and use of vitamin B/multi-vitamin supplements.

## Discussion

The main findings of the present study are that (1) neither dietary cobalamin intake nor the use of supplements are predictors of serum cobalamin after considering other covariables, whereas serum folate and diseases, such as IBD and cancer, are main predictors and (2) serum cobalamin is associated with plasma homocysteine independent of folate and vitamin B_6_ status in community-dwelling older adults.

At present, there is no generally accepted cut-off value with regard to adequate serum cobalamin concentrations. For the diagnosis of cobalamin deficiency, the cut-off of 148 pmol/L has shown a good sensitivity [15, 23] and therefore was applied in our study. Although serum holo-transcobalamin is regarded as the most specific biomarker to characterize adequate cobalamin status, uniform cut-off values are missing [15]. However, concentrations of holotranscobalamin and cobalamin showed a comparable performance as regards screening of subjects for cobalamin deficiency [24].

In the present study, a significant number of subjects had low serum cobalamin (< 148 pmol/L), which could not be explained by low intakes. A similar finding was reported in younger women [25]. Despite the wide range of dietary cobalamin intake in our study (1.2 to 28.2 μg/day), no association with serum cobalamin was found. Other studies reported dietary cobalamin intake to be significantly associated with serum cobalamin [25–27], however, due to the differences in study design and populations, the comparison of the studies is limited. The absorption of cobalamin differs by food source and ingested amount of cobalamin with foods rich in cobalamin, such as liver, showing lower absorption rates of cobalamin than foods containing lower amounts of cobalamin, such as milk, fish and eggs [28, 29]. In the Hordaland Homocysteine Study, cobalamin intake from dairy products led to the greatest increase in plasma cobalamin in comparison to cobalamin intake from other food sources [30]. Although the overall consumption of milk/milk products correlated with cobalamin intake and serum cobalamin before adjustments in our study, cobalamin intake via milk/milk products was not associated with serum cobalamin after considering relevant covariables.

Likewise, the use of vitamin B/multi-vitamin supplements did not predict serum cobalamin after adjustments for other covariables. The same was true when the use of cobalamin supplements within 3 days before examination was analyzed as potentially predicting variable (data not shown). As described earlier, the amounts of cobalamin taken via supplements were rather modest in the GISELA study [19]. Normally, a cobalamin intake of 1.5–2 µg per meal results in saturation of the intestinal absorption via intrinsic factor [31]. Furthermore, the associations between cobalamin intake and status parameters seem to reach a plateau at daily cobalamin intakes around 4–7 µg [26, 27, 32]. Thus, the lacking association of cobalamin intake by diet and supplements with serum cobalamin in our cohort may be due to the fact, that the overall cobalamin intake was sufficient.

In the present study, serum folate was the main predictor of serum cobalamin. Significant correlations of serum/plasma folate with serum/plasma cobalamin were reported in other studies [33–36]. While the metabolic interaction of folate and cobalamin with regard to the transfer of methyl groups is well understood and cobalamin deficiency can lead to an increase in serum folate [37], less is known about possible mechanisms that could explain the pronounced positive association between both vitamins and therefore this deserves further investigation.

In our cohort, besides serum folate, only IBD and cancer proved as robust predictors of serum cobalamin. Cobalamin and folic acid deficiencies are common in patients with IBD for which reason cobalamin preparations may be advised or given parenterally [38], what may explain the positive association of IBD and serum cobalamin in our study. In contrast, we found a negative association of cancer and serum cobalamin. A low cobalamin status has been reported in patients with cancer of the gastrointestinal tract [39] and multiple myeloma [40]. Whether dietary factors, malabsorption and/or metabolic alterations are involved in this relationship requires further exploration.

In the present study, sex, body composition and lifestyle factors were no predictors of serum cobalamin and the initially observed positive effect of age vanished in sub-analyses. Thus, we cannot confirm an independent negative effect of age on serum cobalamin in community-dwelling older adults.

Despite higher cobalamin intakes, male subjects had lower serum cobalamin before adjustments. This was also reported in an elderly population in the Netherlands [26]. One explanation could be differences in distribution volume, which may be higher in males due to higher body mass and FFM compared to females. However, we found no association between FFM and cobalamin status and also no association between serum creatinine and serum cobalamin. Serum creatinine was predominantly within reference range and then may rather reflect muscle mass than kidney function [41].

Inverse correlations between current/past smoking and serum cobalamin were reported in some studies [35, 42], while others found no such relation [43] or even higher serum cobalamin in smokers [44]. Before adjustments, we observed lower serum cobalamin in current/past smokers in the entire cohort. This was not the case in sex-specific analysis what may be explained by the fact that male subjects, who represented the vast majority of smokers, had lower serum cobalamin.

Serum cobalamin was a negative predictor of plasma homocysteine before and after multiple adjustments including serum folate and PLP. We are not aware of any other observational study showing an independent effect of serum cobalamin on homocysteine in community-dwelling older subjects having mostly cobalamin intakes and serum concentrations within reference ranges. A meta-analysis of studies on effects of folic acid supplementation with or without cobalamin on homocysteine levels showed that the addition of cobalamin resulted in additional reductions of homocysteine beyond those due to folic acid [45]. In another study, folic acid supplementation with increasing doses was associated with a decrease in the homocysteine lowering effect, while cobalamin then became a core determinant of plasma homocysteine [46]. Although both folate and cobalamin are required for the remethylation of homocysteine to methionine, the mechanism by which the dependency of the homocysteine lowering effect on cobalamin increases at higher folate intakes is not known. Possible, genetic factors may play a role such as methylentetrahydrofolate reductase and cobalamin dependent polymorphisms [47].

Some limitations must be considered when interpreting the results of this study. Due to the cross-sectional design reverse causality cannot be excluded. We rely on one biomarker to assess cobalamin status and could not include genetic profiles in our assessment. Because of the low prevalence of IBD and cancer, these results should be interpreted with caution. As strengths of our study, we want to point out that the participants were community-dwelling subjects mainly without cobalamin deficiency and that folate, vitamin B_6_, serum creatinine and body composition were considered as relevant covariables.

In conclusion, our results emphasize that in community-dwelling older adults neither cobalamin intake nor age *per se* are significant predictors of serum cobalamin, whereas diseases, such as IBD or cancer, may affect cobalamin status. The underlying mechanisms of the strong positive association between serum folate and cobalamin, as well as the robust independent negative association between serum cobalamin and homocysteine warrant further research.
